# Surveillance or no surveillance for deep venous thrombosis and outcomes of critically ill patients: A study protocol and statistical analysis plan

**DOI:** 10.1097/MD.0000000000012258

**Published:** 2018-09-07

**Authors:** Yaseen M. Arabi, Karen E. A. Burns, Fahad Al-Hameed, Sami Alsolamy, Mohammed Almaani, Yasser Mandourah, Ghaleb A. Almekhlafi, Ali Al Bshabshe, Mohammed Alshahrani, Imran Khalid, Hassan Hawa, Zia Arshad, Hani Lababidi, Abdulsalam Al Aithan, Jesna Jose, Sheryl Ann I. Abdukahil, Lara Y. Afesh, Abdulaziz Al-Dawood

**Affiliations:** a Intensive Care Department, College of Medicine-Riyadh, King Saud Bin Abdulaziz University for Health Sciences, King Abdullah International Medical Research Center, Riyadh, Kingdom of Saudi Arabia; b Interdepartmental Division of Critical Care Medicine, St Michael's Hospital, Li Ka Shing Knowledge Institute, Toronto, Canada; c Department of Intensive Care, College of Medicine-Jeddah, King Saud bin Abdulaziz University for Health Sciences, King Abdullah International Medical Research Center, Jeddah; d Intensive Care and Emergency Medicine Departments, College of Medicine-Riyadh, King Saud Bin Abdulaziz University for Health Sciences, King Abdullah International Medical Research Center; e Department of Pulmonary & Critical Care Medicine King Fahad Medical City, King Saud Bin Abdulaziz University for Health Sciences; f Department of Intensive Care Services, Prince Sultan Military Medical City, Riyadh; g Department of Critical Care Medicinem, King Khalid University, Asir Central Hospital, Abha; h Department of Emergency and Critical Care, Imam Abdulrahman Bin Faisal University, Dammam; i Critical Care Section, Department of Medicine, King Faisal Specialist Hospital & Research Center, Jeddah; j Critical Care Medicine Department, King Faisal Specialist Hospital and Research Centre, Riyadh, Kingdom of Saudi Arabia; k Department of Anesthesiology and Critical Care, King George's Medical University, Lucknow, India; l Department of Pulmonary & Critical Care Medicine, King Fahad Medical City, Riyadh; m Intensive Care and Pulmonary Medicine, King Saud bin Abdulaziz University for Health Sciences, King Abdullah International Medical Research Center, Al Ahsa; n Department Biostatistics and Bioinformatics; o Research Office, King Saud bin Abdulaziz University for Health Sciences, King Abdullah International Medical Research Center, Riyadh, Kingdom of Saudi Arabia.

**Keywords:** critical care, deep vein thrombosis, eligible nonenrolled, intensive care, intermittent pneumatic compression, pulmonary embolism, surveillance, thromboprophylaxis, ultrasound

## Abstract

**Objective:**

Surveillance ultrasounds in critically ill patients detect many deep venous thrombi (DVTs) that would otherwise go unnoticed. However, the impact of surveillance for DVT on mortality among critically ill patients remains unclear.

**Design:**

We are conducting a multicenter, multinational randomized controlled trial that examines the effectiveness of adjunct intermittent pneumatic compression use with pharmacologic thromboprophylaxis compared to pharmacologic thromboprophylaxis alone on the incidence of proximal lower extremity DVT in critically ill patients (the PREVENT trial). Enrolled patients undergo twice weekly surveillance ultrasounds of the lower extremities as part of the study procedures. We plan to compare enrolled patients who have surveillance ultrasounds to patients who meet the eligibility criteria but are not enrolled (eligible non-enrolled patients) and only who will have ultrasounds performed at the clinical team's discretion. We hypothesize that twice-weekly ultrasound surveillance for DVT in critically ill patients who are receiving thromboprophylaxis will have more DVTs detected, and consequently, fewer pulmonary emboli and lower all-cause 90-day mortality.

**Discussion:**

We developed a detailed a priori plan to guide the analysis of the proposed study and enhance the validity of its results.

## Abstract 2

Supplemental Digital Content is available in the text

## 1 Introduction

Venous thromboembolism (VTE), encompassing deep vein thrombosis (DVT) and pulmonary embolism (PE), is associated with substantial morbidity and mortality among patients admitted to the intensive care unit (ICU) even with adequate prophylaxis.^[1]^ DVT is often silent and may result in fatal PE. A systematic review of autopsy-confirmed diagnostic errors in ICU patients demonstrated that 28% of autopsies reported at least one misdiagnosis, with PE being among the leading causes of potentially fatal misdiagnoses.^[2]^ A case-control study demonstrated that DVT risk stratification and history and physical examination performed poorly in discriminating among patients who did and who did not have DVT, indicating that history and physical examination for DVT are not useful in detecting lower limb DVT in the ICU.^[3]^ A retrospective study in hospitalized trauma patients who underwent once weekly surveillance ultrasounds found that 86% of identified DVTs were not clinically suspected.^[4]^

As such, surveillance for DVT has been proposed to detect silent DVT. Several studies have showed that surveillance detects many DVT that would otherwise go unnoticed.^[3]^ Data from recent RCTs in critically ill patients that conducted surveillance for DVT reported much higher DVT incidence that what has been traditionally reported in nonsurveillance studies. For example, the EPO-TBI (Erythropoietin in Traumatic Brain Injury) trial documented a 17% incidence of DVT among patients with traumatic brain injury with once weekly ultrasound.^[5]^ The PROTECT trial (PROphylaxis for ThromboEmbolism in Critical Care Trial) documented 10% incidence of DVT among nontrauma ICU patients with twice-weekly ultrasounds.^[6]^ Similarly, a review of studies that examined DVT in postoperative hospitalized patients showed that surveillance studies reported much higher incidence of DVT than studies that reported symptomatic DVT only.^[7]^

By earlier identification of silent DVTs, surveillance may reduce the incidence of PE and consequently reduce morbidity and mortality in critically ill patients. However, the supportive evidence for this premise in ICU patients is limited. In a pre-post study of implementing a twice-weekly surveillance for DVT in 4234 trauma ICU patients (pre 1422 and post 2812), the rate of DVT diagnosis increased by more than double (odds ratio [OR] 2.53; 95% CI 1.46–4.38) and the rate of PE decreased by about half (OR: 0.49; 95% CI: 0.26–0.90), but there was no change in mortality.^[8]^ As a result of the pre-post design of the study, differences were noted in baseline characteristics including the type and severity of trauma between the 2 time periods. Because of the limited evidence, it remains unclear whether the clinical benefit of DVT surveillance in detecting (and treating) silent DVTs compared to a clinician-directed approach outweighs the risks of anticoagulation and confers benefit on mortality in critically ill patients.

In the Pneumatic CompREssion for Preventing VENous Thromboembolism (PREVENT) trial, we will examine the effectiveness of adjunct intermittent pneumatic compression (IPC) use with pharmacologic thromboprophylaxis compared to pharmacologic thromboprophylaxis alone on the incidence of proximal lower extremity DVT in critically ill patients. Enrolled patients undergo twice weekly surveillance ultrasounds as part of the study procedures. In the proposed analysis, we will compare enrolled patients who will have surveillance ultrasounds to patients who meet the eligibility criteria but are not enrolled (eligible non-enrolled patients or ENE) and who will have ultrasounds performed at the clinical team's discretion. We hypothesize that twice-weekly ultrasound surveillance for DVT in critically ill patients who were receiving thromboprophylaxis will have more DVTs detected, and consequently, fewer pulmonary emboli (PE) and lower all cause 90-day mortality.

## 2 Methods

### 2.1 Setting

The PREVENT trial is a concealed, stratified, unblinded, multicenter, multinational randomized controlled trial that examines the effectiveness of adjunct IPC use with pharmacologic thromboprophylaxis compared to pharmacologic thromboprophylaxis (with unfractionated heparin or low-molecular-weight heparin alone on the incidence of proximal lower extremity DVT in critically ill patients). The trial protocol and statistical analysis plan have been published previously.^[9,10]^ The trial is registered at Clinicaltrials.gov: NCT02040103 and Current controlled trials: ISRCTN44653506. The PREVENT trial is being conducted in 19 sites in Saudi Arabia, Canada, Australia, and India.

### 2.2 Study population

In this analysis, we will include data from participating sites in the PREVENT trial, which have ethics approval to collect minimal dataset on ENE patients and have reported at least 5 ENE cases. The surveillance group includes patients who were enrolled in the PREVENT trial. The nonsurveillance group will include ENE patients except those who decline informed consent and do not give permission for data collection. Patients who are not enrolled for other reasons will be included in the nonsurveillance group including patients who are unable to give consent and no substitute decision-maker is available, patients in whom informed consent is declined but with agreement to collect of minimal observational data, patients who are unable to provide consent within the randomization window of 48 hours from ICU admission, patients who are not enrolled because either the ICU physician or another treating clinician refused enrollment, and patients who were co-enrolled in trials with biologic interaction.

### 2.3 Data collection

We will collate and compare baseline data including: demographics (age, sex), body mass index, location immediately before ICU admission, Acute Physiology and Chronic Health Evaluation (APACHE) II scores, admission categories, and chronic illnesses defined by the APACHE II system between groups.^[11]^ We will also compare predefined pre-ICU VTE risk factors between groups including personal history of VTE, family history of VTE, known thrombophilic states (protein C, protein S, or antithrombin deficiency, thrombotic thrombocytopenic purpura, hemolytic-uremic syndrome, activated protein C resistance, factor V Leiden thrombophilia, prothrombin gene mutation, antiphospholipid antibody, hyperhomocysteinemia), post-partum status (within 3 months), estrogen therapy (oral contraceptive or hormone replacement), active malignancy (treatment within the past 6 months or palliation), history of malignancy (within the past 5 years, other than non-melanoma skin cancer), paralysis or immobilization of a lower or upper extremity related to stroke or injury before this hospital admission, hospitalization in the past 3 months for any reason (excluding this hospital admission), trauma (including acute spinal cord injury, hip fracture, pelvic fracture, femoral fracture and tibial, fibular, knee or other fractures below knee), recent surgery (in the last 48 h), and acute stroke (in this index hospital admission). We will compare baseline platelet count, international normalized ratio, partial thromboplastin time, and serum creatinine.

We will note the type of pharmacologic prophylaxis received by the 2 groups at the time of admission and the presence of femoral central venous catheter at the time of screening. We will also record and compare data on DVT prophylaxis including IPC use (for at least 1 day), graduated compression stockings (GCS) application (for at least 1 day) and therapeutic anticoagulation during ICU stay. The number of all radiologic tests performed for VTE detection during ICU stay will be recorded including lower extremity ultrasound, upper extremity and neck ultrasound, spiral computed tomography (CT) (CT angiogram or helical CT scan) to evaluate for PE, ventilation-perfusion (V/Q) scan of the lungs, CT scan of the abdomen to evaluate thrombosis, transthoracic echocardiogram and trans-esophageal echocardiogram. We will record outcomes including lower extremities DVT, PE, the duration of mechanical ventilation, ICU and hospital length of stay (LOS), and mortality. The primary outcome of the PREVENT trial will be all cause 90-day mortality.

### 2.4 Statistical analysis plan

Categorical variables will be reported as numbers and frequencies, and will be compared using the *χ*^2^ test. Continuous variables will be reported as mean and standard deviation or median and interquartile ranges (IQR, Q1–Q3). Continuous variables will be tested using the Student *t* test or the Wilcoxon-Mann-Whitney test, as judged appropriate by normality testing. Because the assignment to surveillance and nonsurveillance group is not random, we will assess the association of the exposure (surveillance) and time to hospital mortality using multivariate logistic regression and Cox proportional hazards models analysis adjusting for Acute Physiology and Chronic Health Evaluation (APACHE) II score, type of pharmacologic prophylaxis received, presence of femoral central venous catheter, and use of IPC and GCS. We will include also other variables that are significantly different between the 2 groups (*P* < 0.1). Stratified analysis will be performed based on type of admission (medical versus others), type of pharmacologic prophylaxis (UFH vs. LMWH), and IPC use. Interaction terms will be included to assess effect modification of the subgroups on the association between the exposure and outcome. Assuming that we enroll 6 of each 7 eligible patients (6 enrolled patient: 1 eligible non-enrolled patient) and recognizing that the majority of participating centers have approval to collect data on ENE patients, we expect that 90% of the 2000 patients who will be enrolled in the PREVENT trial will be included in the proposed analysis (projected enrollment of 1800 with 300 ENE patients). Anticipating that the mortality of enrolled patients is 20%, our study will have 80% power at 0.05 alpha to detect 7.5% higher mortality in the nonsurveillance group. All statistical analyses will be conducted using the SAS software version 9.1.3 or higher (SAS Institute, Cary, NC).

## 3 Discussion

The proposed substudy addresses the question of whether surveillance ultrasound in critically ill patients by facilitating DVT detection reduces the incidence of PE and lowers all cause 90-day mortality.

Our study has several strengths. The multicenter and multinational nature of the trial enhances the generalizability of its findings. The prospective nature of data collection improves data validity. As per the study protocol, ultrasounds are being performed by certified ultrasound technicians and interpreted by radiologists, which increases the accuracy of the results. Although the present study is not randomized, the 2 groups are run in parallel in the context of a large randomized trial, which reduces the risk of bias related to the use of historical controls that have been used in pre-post studies previously. We will adjust for relevant covariates, if noted, that differ between groups.

However, our study also has limitations. First, as it is not randomized, the presence of unmeasured confounders cannot be entirely excluded. Second, we will plan to conduct a cost-effective analysis based on the findings. To this end, one study showed that implementing surveillance for DVT in trauma ICU patients was associated with incremental costs that compare well with other life-saving interventions that have been accepted by the critical care community.^[8]^ A decision analytic model that compared DVT surveillance to a case finding approach demonstrated increased DVT detection and a reduction in subsequent VTE events. However, surveillance for DVT was associated with more bleeding events caused by a greater frequency of anticoagulation and a higher number of false-positive DVTs detected. This study found that the quality-adjusted survival was improved minimally and could not justify the additional costs of DVT surveillance compared with other commonly used interventions in critically ill patients.^[12]^ Finally, because we use ultrasounds performed by certified technicians and radiologists, the proposed study cannot clarify whether the same results could be achieved with point of care ultrasound performed by intensivists at the bedside.

In conclusion, this article outlines a priori plans for study protocol and analysis plan for surveillance or no surveillance for DVT and outcomes of critically ill patients.

## Acknowledgments

PREVENT sites, principal investigator, co-investigator(s) and research coordinator(s).

The PREVENT trial Group

Saudi Arabia

Assir Central Hospital, Abha: Dr. Ali Al Bshabshe, Dr. Abdulmoniem Albahar, Dr. Ali Alamri

King Abdulaziz Hospital, Ahsa: Dr. Abdulsalam Al Aithan, Shahinaz Bashir

King Abdulaziz Medical City, Jeddah: Dr. Fahad Al-Hameed, Dr. Gulam Rasool, Dr. Jalal Rifai, Ali S. Mohamed, Ohoud Al Orabi

King Abdulaziz Medical City, Riyadh: Dr. Yaseen Arabi, Dr. Abdulaziz Al-Dawood, Dr. Sami Alsolamy, Dr. Mohamed Hegazy, Dr. Maamoun Dbsawy, Sheryl Ann I. Abdukahil, Lara Afesh

Imam Abdulrahman Bin Faisal University, Dammam: Dr. Mohammed Alshahrani, Laila Perlas Asonto, Kathrina Libunao-de Loyola,

King Fahad Medical City: Dr. Mohammed Almaani, Dr. Hani Lababidi, Dr. Husain Abdulmuthalib, Pendo Ntinika, Rachelle Pangilinan

King Faisal Specialist Hospital and Research Center, Jeddah: Dr. Imran Khalid, Dr. Ismael Qushmaq, Dr. Abdulaziz Jadkareem, Eman Bawazeer, Sawsan Bassi, Maryam Imran, Manahil Imran

King Faisal Specialist Hospital and Research Center, Riyadh: Dr. Hassan Hawa, Dr. Khalid Maghrabi, Dr. Mohammad Hijazi, Dr. Musaab Abdelhai, Ellen Joy Pagunsan, Marketa Vinklerova

Prince Sultan Military Medical City: Dr. Yasser Mandourah, Dr. Ghaleb Almekhlafi, Dr. Dina Al Sufiani, Dr. Emad Al Amodi, Dr. Osama El Faki, Shatha Awad, Ma. Raylin Cubio Cabal, Jean S. Valerio, Dr. Sahar Hassan

Canada

Saint Michael's Hospital: Dr. Karen Burns, Orla Smith, Marlene Santos, Gyan Sandhu, Jennifer Hodder, Kurtis Salway, Imrana Khalid, Muhammad Abdul Rehman

India

King George's Medical University: Dr. Zia Arshad, Sachin Kumar Srivastava, Avinash Singh

## Author contributions

Y.A.: Conception and design, analytical plan, interpretation of data for the work, drafting of the manuscript, critical revision of the manuscript for important intellectual content, approval of the final version to be published and agreement to be accountable for all aspects of the work.

K.B., F.H., S.S., M.A., Y.M., G.M., A.B., M.S., I.K., H.H., Z.I., H.L., A.A., J.J., S.A., L.A., A.D.: Data acquisition, critical revision of the manuscript for important intellectual content, approval of the final version to be published and agreement to be accountable for all aspects of the work.

**Conceptualization:** Yaseen Arabi.

**Data curation:** Yaseen Arabi, Karen Burns, Fahad Al-Hameed, Sami Alsolamy, Mohammed Almaani, Yasser Mandourah, Ghaleb Almekhlafi, Ali Al Bshabshe, Mohammed Alshahrani, Imran Khalid, Hassan Hawa, Zia Arshad, Hani Lababidi, Abdulsalam Al Aithan, Jesna Jose, Sheryl Ann Abdukahil, Lara Afesh, Abdulaziz Al-Dawood.

**Formal analysis:** Yaseen Arabi.

**Funding acquisition:** Yaseen Arabi.

**Methodology:** Yaseen Arabi, Karen Burns.

**Validation:** Jesna Jose.

**Writing – original draft:** Yaseen Arabi, Karen Burns.

**Writing – review & editing:** Yaseen Arabi, Karen Burns, Fahad Al-Hameed, Sami Alsolamy, Mohammed Almaani, Yasser Mandourah, Ghaleb Almekhlafi, Ali Al Bshabshe, Mohammed Alshahrani, Imran Khalid, Hassan Hawa, Zia Arshad, Hani Lababidi, Abdulsalam Al Aithan, Jesna Jose, Sheryl Ann Abdukahil, Lara Afesh, Abdulaziz Al-Dawood.

## Supplementary Material

Supplemental Digital Content
