# Supplementary material for: Surveillance or no surveillance for deep venous thrombosis and outcomes of critically ill patients: A study protocol and statistical analysis plan
Source: Medicine (Baltimore). 2018 Sep 7;97(36):e12258. doi: 10.1097/MD.0000000000012258 (PMC6133421; doi:10.1097/MD.0000000000012258)
Supplement: Supplemental Digital Content [file medi-97-e12258-s001.docx]

| **Table S1:** Baseline characteristics. |  |  |
| --- | --- | --- |
| **Characteristic** | **Surveillance Group(N=XXX)** | **No Surveillance Group (N=XXX)** |
| Age (Years) - Mean (SD) | xx (xx.x) | xx (xx.x) |
| Male sex - n (%) | xxxx/xxxx (xx.x) | xxxx/xxxx (xx.x) |
| Height (cm) - Mean (SD) | xx (xx.x) | xx (xx.x) |
| Weight (kg) - Mean (SD) | xx (xx.x) | xx (xx.x) |
| BMI (kg/m^2^) - Mean (SD) | xx (xx.x) | xx (xx.x) |
| Location prior to ICU admission - n (%) |  |  |
| Emergency room | xxxx (xx.x) | xxxx (xx.x) |
| Hospital ward | xxxx (xx.x) | xxxx (xx.x) |
| Operating room | xxxx (xx.x) | xxxx (xx.x) |
| Other hospital (ICU or ward) | xxxx (xx.x) | xxxx (xx.x) |
| Other | xxxx (xx.x) | xxxx (xx.x) |
| APACHE II- Mean (SD) | xx (xx.x) | xx (xx.x) |
| Chronic Health Illnesses - n (%) |  |  |
| None | xxxx (xx.x) | xxxx (xx.x) |
| Liver disease | xxxx (xx.x) | xxxx (xx.x) |
| Cardiovascular disease | xxxx (xx.x) | xxxx (xx.x) |
| Respiratory disease | xxxx (xx.x) | xxxx (xx.x) |
| Renal disease | xxxx (xx.x) | xxxx (xx.x) |
| Immunosuppression | xxxx (xx.x) | xxxx (xx.x) |
| Pre-ICU conditions that may influence VTE risk - n (%) |  |  |
| Personal history of VTE | xxxx (xx.x) | xxxx (xx.x) |
| Family history of VTE | xxxx (xx.x) | xxxx (xx.x) |
| Known thrombophilic state | xxxx (xx.x) | xxxx (xx.x) |
| Post-partum (within 3 months) | xxxx (xx.x) | xxxx (xx.x) |
| Estrogen therapy | xxxx (xx.x) | xxxx (xx.x) |
| Active malignancy (treatment within past 6 months or palliation) | xxxx (xx.x) | xxxx (xx.x) |
| History of malignancy (past 5 years; other than non-melanoma skin cancer) | xxxx (xx.x) | xxxx (xx.x) |
| Paralysis or immobilization of a lower or upper extremity related to stroke or injury  prior to this hospital admission | xxxx (xx.x) | xxxx (xx.x) |
| Hospitalization in the past 3 months for any reason (excluding this hospital admission) | xxxx (xx.x) | xxxx (xx.x) |
| Trauma | xxxx (xx.x) | xxxx (xx.x) |
| Recent surgery (in the last 48 hours) | xxxx (xx.x) | xxxx (xx.x) |
| Acute stroke (this hospital admission) | xxxx (xx.x) | xxxx (xx.x) |
| Others | xxxx (xx.x) | xxxx (xx.x) |
| None | xxxx (xx.x) | xxxx (xx.x) |
| Laboratory results prior to randomization - Mean (SD) |  |  |
| INR (highest) | xx (xx.x) | xx (xx.x) |
| Creatinine (µmol/L) | xx (xx.x) | xx (xx.x) |
| Platelets (10^9^/L) | xx (xx.x) | xx (xx.x) |
| PTT (highest) | xx (xx.x) | xx (xx.x) |
| Hemoglobin (g/L) | xx (xx.x) | xx (xx.x) |
| Central Venous Lines |  |  |
| Femoral- n (%) | xxxx (xx.x) | xxxx (xx.x) |

**APACHE**- Acute Physiology and Chronic Health Evaluation; **INR**- International normalized ratio; **PTT**- Partial thromboplastin time; **SD**-standard deviation; **VTE**- Venous Thromboembolism

| **Table S2:** Summary of interventions and co-interventions. | | |
| --- | --- | --- |
| **Variable** | **Surveillance Group (N=XXX)** | **No Surveillance Group (N=XXX)** |
| Use of IPC |  |  |
| Number of patients receiving IPC at least for one day | xxxx (xx.x) | xxxx (xx.x) |
| Pharmacologic prophylaxis at the time of enrollment |  |  |
| Prophylactic UFH | xxxx (xx.x) | xxxx (xx.x) |
| Prophylactic LMWH | xxxx (xx.x) | xxxx (xx.x) |
| Diagnostic Testing, n (%) |  |  |
| Lower extremities ultrasonography |  |  |
| Patients with at least one ultrasonography – n (%) | xxxx (xx.x) | xxxx (xx.x) |
| Ultrasonography for upper extremities and neck to evaluate for thrombosis – n (%) | xxxx (xx.x) | xxxx (xx.x) |
| Patients with spiral CT (also called CT angiograms or helical CTscan) of chest to evaluate for PE– n (%) | xxxx (xx.x) | xxxx (xx.x) |
| Patients with V/Q scan of the lungs– n (%) | xxxx (xx.x) | xxxx (xx.x) |
| Patients with CT scan of the abdomen to evaluate thrombosis– n (%) | xxxx (xx.x) | xxxx (xx.x) |
| Patients with transthoracic echocardiograms– n (%) | xxxx (xx.x) | xxxx (xx.x) |
| Patients with transesophageal echograms– n (%) | xxxx (xx.x) | xxxx (xx.x) |

**IPC**-Intermittent Pneumatic Compression; **LMWH**-Low Molecular Weight Heparin; **PE**-Pulmonary Embolism; **UFH**-Unfractionated heparin

| **Table S3:** Outcomes |  |  |  |  |
| --- | --- | --- | --- | --- |
| **Variable** | **Surveillance Group (N=XXX)** | **No Surveillance Group (N=XXX)** | **aOR, (95% CI)**  **or correlation coefficeient** | **P-value** |
| DVT– n/N (%) | xxxx (xx.x) | xxxx (xx.x) | x.xx (x.xx , x.xx) | x.xxx |
| Pulmonary Embolism– n/N (%) | xxxx (xx.x) | xxxx (xx.x) | x.xx (x.xx , x.xx) | x.xxx |
| ICU mortality – n (%) | xxxx (xx.x) | xxxx (xx.x) | x.xx (x.xx , x.xx) | x.xxx |
| ICU LOS – days | xx (xx.x) | xx (xx.x) | x.xx (x.xx , x.xx) | x.xxx |
| Hospital Mortality – n (%) | xxxx (xx.x) | xxxx (xx.x) | x.xx (x.xx , x.xx) | x.xxx |
| 90-day Mortality – n (%) | xxxx (xx.x) | xxxx (xx.x) | x.xx (x.xx , x.xx) | x.xxx |
| Hospital LOS – days | xx (xx.x) | xx (xx.x) | x.xx (x.xx , x.xx) | x.xxx |

Denominator of the percentage is the total number of subjects in each group.

**DVT**: Deep Vein Thrombosis; **PE**: Pulmonary Embolism; **LOS**: Length of Stay

| **Table S5:** Subgroup analyses for hospital mortality. | | | | | |
| --- | --- | --- | --- | --- | --- |
|  | **Surveillance Group (N=XXX)** | **No Surveillance Group (N=XXX)** | **aOR (95% CI)** | **P-value** | **P-value for interaction** |
| UFH | xxxx/xxxx (xx.x) | xxxx/xxxx (xx.x) | x.xx (x.xx, x.xx) | x.xxx | x.xxx |
| LMWH | xxxx/xxxx (xx.x) | xxxx/xxxx (xx.x) | x.xx (x.xx, x.xx) | x.xxx |  |
| Femoral CVC at baseline | xxxx/xxxx (xx.x) | xxxx/xxxx (xx.x) | x.xx (x.xx, x.xx) | x.xxx | x.xxx |
| No Femoral CVC at baseline | xxxx/xxxx (xx.x) | xxxx/xxxx (xx.x) | x.xx (x.xx, x.xx) | x.xxx |  |
| Trauma | xxxx/xxxx (xx.x) | xxxx/xxxx (xx.x) | x.xx (x.xx, x.xx) | x.xxx | x.xxx |
| Post-operative | xxxx/xxxx (xx.x) | xxxx/xxxx (xx.x) | x.xx (x.xx, x.xx) | x.xxx |  |
| Medical | xxxx/xxxx (xx.x) | xxxx/xxxx (xx.x) | x.xx (x.xx, x.xx) | x.xxx |  |
| BMI <30 | xxxx/xxxx (xx.x) | xxxx/xxxx (xx.x) | x.xx (x.xx, x.xx) | x.xxx | x.xxx |
| BMI >30 | xxxx/xxxx (xx.x) | xxxx/xxxx (xx.x) | x.xx (x.xx, x.xx) | x.xxx |  |
| Saudi Arabia | xxxx/xxxx (xx.x) | xxxx/xxxx (xx.x) | x.xx (x.xx, x.xx) | x.xxx | x.xxx |
| Canada | xxxx/xxxx (xx.x) | xxxx/xxxx (xx.x) | x.xx (x.xx, x.xx) | x.xxx |  |
| India | xxxx/xxxx (xx.x) | xxxx/xxxx (xx.x) | x.xx (x.xx, x.xx) | x.xxx |  |

**BMI:** Body Mass Index; **CVC**: Central venous catheter; **LMWH**-Low Molecular Weight Heparin; **PE**-Pulmonary Embolism; **UFH**- Unfractionated heparin
